# Perceived Social Support from Significant Others among Binge Drinking and Polyconsuming Spanish University Students

**DOI:** 10.3390/ijerph16224506

**Published:** 2019-11-15

**Authors:** Carolina Tinajero, Fernando Cadaveira, M. Soledad Rodríguez, M. Fernanda Páramo

**Affiliations:** 1 Department of Developmental and Educational Psychology, Faculty of Psychology, C/ Xosé María Suárez Núñez, s/n, Campus Vida, 15782 Santiago de Compostela, Spain; mfernanda.paramo@usc.es; 2 Department of Clinical Psychology and Psychobiology, Faculty of Psychology, C/ Xosé María Suárez Núñez, s/n, Campus Vida, 15782 Santiago de Compostela, Spain; fernando.cadaveira@usc.es; 3 Department of Social, Basic Psychology and Methodology, Faculty of Psychology, C/ Xosé María Suárez Núñez, s/n, Campus Vida, 15782 Santiago de Compostela, Spain; msoledad.rodriguez@usc.es

**Keywords:** alcohol drinking, binge, cannabis, university students, social support, adjustment

## Abstract

Sense of acceptance is conceived as a central component of perceived social support and is thought to be a key resilience factor for adjustment during transition to university. The current study examines how a binge drinking pattern of alcohol consumption and the co-consumption of binge drinking and cannabis in first-year university students are related to perceived acceptance from family, mother, father, and friends. The study sample consisted of 268 women and 216 men, of average age 18.25 years (SE = 0.01), enrolled in the first year of different degree courses at the University of Santiago de Compostela. Participants were classified in three groups (control, binge drinking, polyconsuming) on the basis of the Timeline Followback for alcohol and cannabis. Perceived sense of acceptance was measured using the Perceived Acceptance Scale. Analysis of the data revealed that perceived acceptance was lower in polyconsuming students than in the binge drinking and control groups (*p* < 0.05; with η^2^ ranging between 0.009 and 0.020). A curvilinear relationship between binge drinking and perceived acceptance from friends was identified. Social support should be considered in future investigations and interventions as a vulnerability marker for detrimental consequences of substance use and risk of consumption disorders, as well as adolescent maladjustment.

## 1. Introduction

Drug use by young adults is a matter of great concern in most countries due to its negative impact on health and welfare [1,2]. In particular, binge drinking (BD) has been receiving increasing attention as a highly risky pattern of alcohol consumption [3,4,5]. BD is defined as the consumption of four or more drinks for women and five or more drinks for men in about 2 h, leading to a blood alcohol concentration (BAC) of 0.08 g/dL [6].

The currently available survey data reveal that BD is a prevalent pattern of alcohol consumption [2], although rates vary widely among different countries. In the USA, an estimated 38.4% of young adults aged 18 to 25 years are binge drinkers [7]. In European countries, the mean proportion of frequent binge drinkers aged 15 years or above ranges from 11% to 44%, and the global proportion for the youngest age group (15–24 years) is 33% [8]. In Spain, the results of the most recent national survey on drug use [9] have indicated that the prevalence of BD among the population of ages between 14 and 18 years is 31.7%. Indeed, alcohol consumption is culturally tolerated during the transition to university and is often considered to function as a rite of passage to adulthood [10]. 

Various consequences of alcohol consumption have been identified in young people, and BD [11,12,13,14] in particular is thought to have more serious consequences than regular consumption [15]. Thus, young adult binge drinkers are more likely than non-bingers to have physical problems (e.g., hangover, nausea, and vomiting), problems with authorities (e.g., school administration, police), academic problems (e.g., missing class, lower grades) and social problems (e.g., disruption of family relationships, arguing with friends) and to engage in risky behavior (e.g., unplanned sexual activity, drinking and driving) [3,5,16].

In addition, binge drinkers are more likely to use other types of drugs [17,18,19,20]. Cannabis is the most commonly consumed illicit drug [7,9], and cannabis plus alcohol is the most commonly consumed combination of drugs [21]. In Spain, four out of ten binge drinkers of ages between 14 and 18 years declare having used cannabis in the same period as binge drinking [9], which is consistent with data on the co-use of alcohol and cannabis from other countries [22,23,24,25]. Polyconsumption increases the quantity and frequency of cannabis and alcohol use [26,27,28] as well as the risk and severity of detrimental effects [24,29].

The most frequent consequences of binge drinking and polyconsumption affect the social domain [25,30,31,32]. This type of harm has been considered inherently interactional, as it entails problematic behavior on the part of the consumer as well as a reaction by someone else [33]. Some issues typically considered within this category include neglected obligations, disruption of family relations, arguments with or bad feelings between the consumer and family members or friends and doing or saying something that was latter regretted or caused shame or embarrassment [4,15,34].

These effects are likely to alter relationship networks, affecting perceived access to social support resources and even perceived possibilities of providing support [35,36]. Perceived social support has been found to act as a key protective factor in the face of challenging or stressful situations [37,38]. Sense of acceptance has been regarded as the central component of perceived social support and it is defined as a relatively stable cognitive impression that significant others are concerned about us and value us [39]. In addition, the findings of studies with university students suggest that perceived acceptance mediates relationships between other facets of social support and social and academic adjustment [39,40]. 

The negative effects of binge drinking and polyconsumption on perceived social support are of particular interest, given the role of this dimension as a resilience factor for adjustment. Various studies across the world in the last few decades have identified perceived social support as a protective, empowering factor that is key to enabling emerging adults to fulfill the challenges involved in university life [40,41,42]. Notwithstanding, the relationship between substance consumption and perceived social support among university students has scarcely been explored. Regarding alcohol consumption, a few studies have examined perceived support as a correlate of frequency and amount of drink, with contradictory results [43,44,45,46]. Other studies have examined the predictive value of perceived social support in relation to drinking, under the assumption that perceived social support may act as a protective factor against risk behaviors, but no statistically significant effect has been found [47,48,49]. To our knowledge, the reverse directional relationship has not been explored in young adults.

Some evidence has been provided for a U-shaped relationship between binge drinking, peer intimacy, and social integration among university students (identified either by quadratic regression models or by comparisons among groups characterized by different rates of drinking) [50,51,52], indicating that abstainers and low- and high-frequency binge drinkers would have poorer peer relationships than moderate-frequency binge drinkers. Nevertheless, the possible curvilinear relationship between consumption and perceived social support from peers remains unexplored.

There is no information available about the effect of cannabis consumption on perceived social support, with the exception of data obtained in a co-twin study with individuals aged between 21 and 62.5 years [53]. Given that regression coefficients were similar for cannabis use as predictor of perceived support for monozygotic and dizygotic twins and for the average population, the authors concluded that common shared genetic predisposition or environmental factors did not contribute to the relationship, and a causal relationship between consumption and perceived social support may be assumed. 

Finally, in a study investigating the relationship between the combined use of alcohol and other drugs and perceived social support, adolescents aged 14 to 18 years were monitored during 4 years after treatment for alcohol and drug abuse [54]. Individuals who engaged in many more episodes of alcohol or drug use reported less support than abstainers and those who engaged in limited use. We may thus expect a detrimental social effect of the concurrence of binge drinking and cannabis consumption among young adults. 

Further research is needed to elucidate the social consequences of binge drinking and polysubstance use in young people. In particular, perceived social support may be affected, placing individuals who are transitioning to university in a vulnerable position for adjustment. The aforementioned evidence on the social consequences of consumption suggests a possible directional effect of binge drinking and the combined use of cannabis and alcohol on perceived social support. Binge drinking and polyconsumption could plausibly alter the social network of the young students, affecting perceived acceptance from significant others. Some available data from longitudinal [32], co-twin [53] and intervention [54] studies on substance use are consistent with this assumption, although the investigation along this line is still scarce.

The current study aimed to assess and compare perceived acceptance from different sources of social support among non- or sporadically consuming university students and those who report binge drinking or co-consumption of alcohol and cannabis. Consistent with previous findings on how alcohol use and polyconsumption are related to social problems and perceived social support, the following hypotheses were proposed: (1) non- or sporadically consuming university students would perceive greater acceptance from their family, mother, father and friends than their binge drinking and polyconsuming colleagues; (2) binge drinking students would perceive greater acceptance than polyconsumers; and (3) moderate binge drinkers would manifest higher perceived acceptance from friends than non-consumers and low and high binge drinkers.

## 2. Materials and Methods 

### 2.1. Participants and Procedure

A cross-sectional design was conducted within the framework of a cohort study. The study population was composed of first-year students (18–19 years old) attending the University of Santiago de Compostela (*n* = 2998). At least one first-year class was randomly selected from each of the faculties (Santiago Campus). 

The initial data were collected by self-completion questionnaires distributed to the students during classes. Two team researchers visited the classes in October 2016 and invited all students present in each class to participate in the study. 

Subjects were informed both verbally and in written format (within the questionnaire) that participation was voluntary, anonymous, and that they could opt out of the study at any time. Those students willing to continue participating in the study provided a phone number at the end of the questionnaire. 

The Galician validated version of the Alcohol Use Disorder Identification Test (AUDIT-C) [55,56] and three questions regarding consumption of medicines, cannabis, and other illicit substances were used to identify respondents with a profile of risky alcohol consumption, polyconsumption, or non- or sporadic consumption. A cut-off score was established for the AUDIT-C according to gender (>3 for women, >4 for men) [6]. Risky cannabis use was defined as consumption at least once monthly [57]. Individuals who were using any other illicit drugs or any psychoactive medication were excluded from the sample.

A total of 484 students (268 women, 216 men) of average age 18.25 years (SE = 0.01) were selected for a later semi-structured interview to establish their recent alcohol and cannabis use and perceived acceptance. A code was then assigned to each participant for the purpose of data confidentiality and to preserve the blind condition of researchers. Direct personal identifiers were not included in the reply/registration forms. Most (85.1%) of the participants were living away from home and the others (14.9%) were living in the family home. The Timeline Followback (TLFB) [58] was administered to the students selected, in order to produce a retrospective calendar-based measure of recent alcohol (previous 180 days) and cannabis (previous 90 days) use. Male students who consumed 6 or more standard drinks and female students who consumed 4 or more standard drinks on a single occasion, at least once in the last 30 days, were classified as binge drinkers (187 students, 38.6%). The polyconsuming group consisted of students who also consumed at least 3 cannabis units in the last 3 months (119 students, 24.5%) and the control group comprised the remaining 178 students (36.8%).

All the individuals participating in the interview phase signed an informed consent form and received financial compensation (10€). The study was approved by the Bioethics Committee of the University of Santiago de Compostela.

### 2.2. Instruments

#### 2.2.1. Consumption Measure

The Timeline Followback (TLFB) [58] provides retrospective self-report estimates of an individual’s daily substance consumption over a specified period in standard units. As memory aids, participants received a calendar with special events marked on it (e.g., holidays, semester events). They were also recommended to use personal diaries to record special events (e.g., birthday, family events), and consumption routines when pertinent (e.g., drinking at weekends, parties). This method has been found to have high test–retest reliability and validity as a measure of alcohol [59] and cannabis [60,61] consumption.

#### 2.2.2. Perceived Acceptance Measure

The Perceived Acceptance Scale (PAS) [62] was used to evaluate the acceptance dimension of perceived social support from significant others. The PAS is a 44-item self-report measure designed to assess perception of acceptance within four specific categories of relationships, family (12 items), mother (10 items), father (10 items), and friends (12 items), with a score range of 10–50 for PAS family and PAS friends, and of 12–60 for PAS mother and PAS father. Sample items include “My parents objected to a number of things I did” or “I am a very important part of the lives of my friends”. Responses are made on a 5-point Likert-type scale ranging from 1 “strongly disagree” to 5 “strongly agree”. In the current study, internal consistency was satisfactory for the total score and for subscale scores, with Cronbach’s alpha coefficients of 0.94 for the total score, 0.89 for PAS family, 0.88 for PAS mother, 0.90 for PAS father and 0.89 for PAS friends.

### 2.3. Data Analysis

Data were analyzed using IBM SPSS version 24 for Windows. Prior to the primary analyses, data were examined for outliers and assumptions of normality. Homogeneity of variance among groups was determined using Box’s test and Levene’s tests. Chi-square tests were used to compare demographic characteristics between groups (gender, mother’s and father’s educational level, socioeconomic status, residence), and ANOVA was applied to compare indicators of substance use (no. days BD and cannabis unit). Parent’s educational level was categorized into 3 groups (low, middle, high) and socioeconomic status was established by job titles grouped in six occupational categories.

Two-way multivariate analysis of variance (3 × 2) was conducted, with consumption group (control, binge drinking, and polyconsuming) and sex (men vs. women) as independent variables and perceived social support from family, mother, father, and peers as dependent variables. This was followed by univariate analyses to determine the effect of consumption group on perceived acceptance. Gender was also included as an independent variable with the intention of exploring how it interacts with substance consumption, given that differences between male and female university students have been observed in relation to perceived support, binge drinking, and cannabis consumption [8,63,64].

A Bonferroni post-hoc test was used to determine statistically significant differences between groups. Partial eta squared (η^2^) was obtained as an indicator of the size of the effect. Differences indicated by the tests were considered significant at *p* < 0.05.

Finally, regression analysis was conducted to determine whether the relationship between number of days of binge drinking and perceived acceptance from friends was curvilinear (i.e., quadratic).

## 3. Results

The characteristics of the groups (control, binge drinking, polyconsuming) are summarized in Table 1. There were no significant differences between groups for demographic variables, with the exception of residence.

In relation to the effect of consumption group on perceived acceptance, significant multivariate differences (Wilks’ Λ = 0.949, *F*(8,950) = 3,129, *p* = 0.002, η*^2^* = 0.026) emerged for consumption. The mean scores on perceived acceptance for binge drinking, polyconsuming and control groups and the results of the ANOVAs are summarized in Table 2. No gender-related differences were found for perceived acceptance from family, mother, father, or friends.

The Bonferroni post-hoc analysis indicated that perceived acceptance from family was higher in the control (*p* = 0.006) and BD (*p* = 0.011) groups than in the polyconsuming group, and perceived acceptance from friends was higher in BD students than in the control group (*p* = 0.027). 

**Figure 1:**
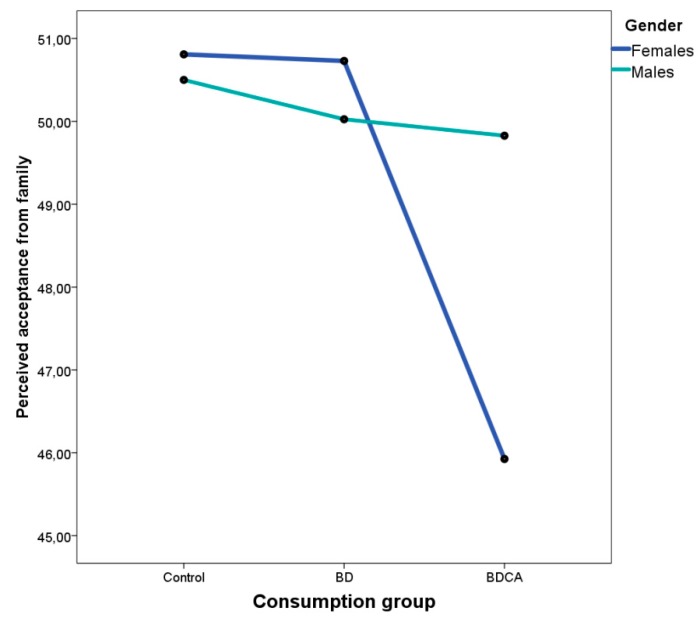
Perceived acceptance from family by consumption group.

The univariate analyses also showed that the interaction between gender and group was significant for perceived acceptance from family (*F*(2,484) = 3.435, *p* = 0.033, η^2^ = 0.014). Figure 1 illustrates this interaction effect, with perceived acceptance from the family being lower for polyconsuming women (*t*(117) = −2.524, *p* = 0.013).

**Figure 2:**
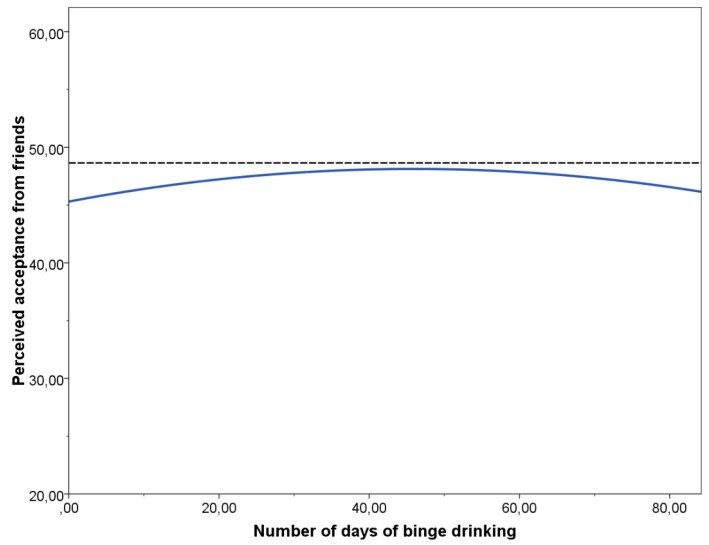
Prediction of perceived acceptance from friends as a function of binge drinking days. Quadratic equation is *Y* = 45.30 + 0.123 *X* − 0.001 *X*^2^.

The expected curvilinear relationship between binge drinking and perceived acceptance from friends was confirmed by the quadratic regression analysis. Thus, the analysis revealed a significant curvilinear relationship between number of days of binge drinking and perceived acceptance from friends (*F* (484) = 3.721, *p* = 0.025, *R^2^* = 0.015), indicating an increase in perceived acceptance until about 30 days of binge drinking (within the previous 180 days), followed by a rather stable level of acceptance until about 50 days of binge drinking, and a decline thereafter (see Figure 2).

## 4. Discussion

The main objective of this study was to assess and compare perceived acceptance from different sources of social support among non- or sporadically consuming university students and students who report binge drinking or co-consumption of alcohol and cannabis. Perceived social support and, in particular, perceived acceptance, have been found to act as key protective factors in the face of challenging or stressful situations [37,38]. They are thought to act by enhancing one’s sense of belonging, self-worth, and security, as well as by moderating the appraisal of situations as threatening and enhancing self-confidence to cope with such situations [37,65]. Based on previous data on the social consequences of substance use among university students, we expected binge drinking consumption to have an effect on perceived acceptance. Specifically, previous studies on the social consequences of binge drinking and polyconsumption have focused on young people in higher education, as both patterns generally peak between the ages of 18 and 25 years [10,66]. At this life stage, when emerging adults are still contending with developmental tasks such as establishing autonomy and personal identity, starting university adds further challenges. First-year students must adapt to a new social environment, manage separation from friends and family and establish relationships with new peer groups while dealing with new responsibilities and academic demands [26,67,68]. Some authors have advised that substance use could impede successful accomplishment of these transitional demands [69,70]. Thus, the effect of alcohol/drug consumption on social support is especially relevant, given the value of social support for adjustment during transition to university.

Non- or sporadically consuming students in the present sample were expected to perceive the highest level of acceptance from their significant others, and polyconsumers were expected to perceive the lowest level of acceptance. Perceived acceptance from the family was indeed significantly higher in the control group and binge drinking groups than in polyconsumers. Thus, co-consumption of alcohol and cannabis seems to negatively affect the students’ sense of being valued and trusted by their families. Different events and circumstances may have contributed to these findings. On the one hand, consumption has been consistently associated with arguments or bad feelings between the consumer and family members [71,72]. The disturbances to family relationships may at least partly correspond to other social and personal consequences of consumption itself, such as neglecting family or academic responsibilities (ignoring housework, missing or being late for class, falling behind in academic work) [15,73].

On the other hand, parent–child interactions often decrease from adolescence into emerging adulthood, and tensions regarding autonomy and connectedness are particularly strong at this stage of development [10,74]. Parents transmit conventional norms and attitudes towards substance use and monitor their children’s health behaviors [74,75,76], while the youngsters pursue independence and try to integrate in the university community and share its culture, which includes alcohol and cannabis use [77,78,79]. Substance consumption may be perceived as a gateway to adulthood, at the expense of losing family acceptance as a result of transgressing family values. 

Moreover, the interaction effects between PAS scores and gender observed in the present study indicate that female polyconsumers perceived lower acceptance from the family than male polyconsumers, plausibly indicating narrower or less tolerant parental monitoring of daughters. Indeed, daughters have previously been found to receive higher levels of parental monitoring than sons [80,81]. 

With regard to perceived acceptance from friends, the most salient finding is that the direction of the relationship with consumption is the opposite of that observed for acceptance from the family. Namely, the control group perceived lower acceptance from friends than the binge drinking group, although differences between the control and polyconsuming groups were not statistically significant. Effects beyond detrimental social consequences of consumption must be considered in interpreting these findings, as binge drinking seems to at least provide some benefit to perceived peer status of university students in the present sample. Subjective positive correlates of drinking in university students have recently been investigated [82,83]. Four types of reinforcing outcomes have been distinguished, namely enhancement (e.g., having fun), coping (e.g., forgetting problems), social (e.g., increasing sociability), and conformity (e.g., fitting with the group) [84]. These reinforcing effects may prevail over detrimental consequences during transition to university. In particular, having fun and social consequences are the reasons most frequently given [82]. Indeed, these types of effects may be particularly salient in first-year students who are trying to organize a new social network and adjust their behavior to perceived peer norms [85]. Increased access to drinking situations and overestimation of peer consumption may also contribute to this supposed appraisal bias [85,86,87]. 

Another result expected in our study was that relative to their colleagues, moderate binge drinking students would perceive higher acceptance from their friends. Indeed, perceived acceptance increased with frequency of heavy drinking, peaked at 30–50 days of binge drinking, and decreased with additional binge drinking episodes. Thus, the results of the present study are consistent with those of previous investigations reporting a curvilinear relationship between binge drinking and quality of peer relationships [50,51,52]. Nevertheless, contrary to previous studies, the present sample only comprised first-year students, and data on their alcohol use were recorded at the beginning of the academic year. The possible decline in acceptance from friends as a result of more frequent binge drinking should be examined further in future studies with older university students, in order to encompass the peak consumption period [63,88].

In summary, the findings of the present study add to our knowledge of the social consequences of binge drinking and co-use of alcohol and cannabis among university students. Consumption seems to have a detrimental effect on the perceived acceptance from the family, probably increasing normative distancing and feelings of isolation due to leaving the parental home and being separated from the peer group and relatives [10]. 

Consuming environments may help to relieve the transition to a new support network by providing opportunities to have fun and make contact and gain acceptance from peers [89,90]. Indeed, our findings suggest that binge drinking actually increases perceived acceptance from friends. This effect may facilitate social adjustment to university [40] but at the same time contribute to perpetuating or worsening consumption patterns as drinking has been shown to affect youngsters’ self-regulation and self control [91,92], and reciprocal influences between drinking and positive consequences of drinking have been observed [93,94]. 

Parents could provide a way out of this vicious circle, as students continue to seek support from the family after leaving home, and communication with parents during college has been found to protect against substance use [45,87,95]. Thus, stimulating parental communication is an essential line of preventive intervention suggested by the present findings. Additionally, young adults’ perceptions and expectations of negative and positive consequences of consumption should be emphasized in intervention proposals, and alternative healthy reinforcing activities should be promoted, particularly among students starting university.

Finally, our findings and interpretations must be considered with caution in view of some limitations of the present study. First, the cross-sectional study design does not allow proposal of a causal order in the relationship between consumption and perceived acceptance. Although the assumption of an effect of binge drinking and polyconsumption on perceived social support is consistent with previous data on detrimental and positive social consequences of consumption, further longitudinal and intervention studies should be performed to establish the possible causal nature of the association between consumption and perceived social support. As a second limitation, the sample included only first-year students, whereas variations in the relationship between consumption and peer acceptance may be expected during all years at university. Third, binge drinking and polyconsuming students were treated as homogeneous groups, and the frequency and intensity of binge drinking as well as the type of polyconsumption (complementary, concurrent, simultaneous) should be taken into account in future studies. Collection of a reliable large data set and implementing a real-time statistical analysis would enable a high impact research on the effects of binge drinking and polyconsumption in students with different profile types, throughout the university years [95,96]. Despite the aforementioned limitations, to our knowledge, this is the first study to examine the relationships between perceived acceptance from significant others and binge drinking and co-occurrence of BD and cannabis. The findings indicate that perceived acceptance should be taken into account as a marker of the problematic status of consumers or of their vulnerability in view of the demands posed by the transition to university.

## 5. Conclusions

The study findings highlight the association between alcohol and cannabis consumption and perceived social support, a dimension that is considered a key protective factor in the face of challenging or stressful situations such as transition to university. The results of the study reveal that perceived acceptance from family was lower among polyconsuming first-year university students than among their binge drinking and non- or sporadically consuming colleagues. A curvilinear relationship between binge drinking and perceived acceptance from friends was observed; relative to their colleagues, moderate binge drinking students perceived higher acceptance from their friends. Social support should be considered as a vulnerability marker for adolescent maladjustment in future studies and interventions.

## Figures and Tables

**Table 1:** Characteristics of the students (% or M and SD) and *p*-value for comparative statistics.

Variable	Control (*n* = 178)	BD (*n* = 187)	BDCA (*n* = 119)	*p*-Value
Gender				ns
Male	47.2	42.8	43.7	
Female	52.8	57.2	56.3	
Mother’s educational level				ns
Primary school	32.2	27.1	25.4	
High school	21.5	26	27.1	
University	46.3	47	47.5	
Father’s educational level				ns
Primary school	36	37.6	30.4	
High school	25.6	23.6	26.1	
University	38.4	38.8	43.5	
Socioeconomic status				ns
Low	12.6	11.5	16.7	
Middle	84	81.9	78.9	
High	3.4	6.6	4.4	
Residence				0.01
In family home	20.8	13.4	8.4	
Away from home	79.2	86.6	91.6	
No. days binge drinking (BD)	1.04 (1.59)	20.96 (12.12)	29.35 (14.59)	<0.001
Cannabis unit	0	0.32 (1.09)	29.36 (60.25)	<0.001

**Table 2:** Means, standard deviations, and ANOVA results for the Perceived Acceptance Scale (PAS) scores by group.

PAS	Control (*n* = 178)	BD (*n* = 187)	BDCA (*n* = 119)	*F*	*p*-Value	η^2^
	M(SD)	M(SD)	M(SD)			
Family	50.663 (7.646)	50.428 (7.916)	47.630 (8.551)	4.927	0.008	0.020
Mother	43.809 (7.029)	43.423 (6.581)	41.706 (7.079)	2.618	0.074	0.011
Father	40.573 (9.143)	41.171 (8.241)	38.782 (9.460)	2.142	0.119	0.009
Friends	45.270 (9.092)	47.631 (7.669)	46.874 (8.402)	3.588	0.028	0.015

BD = binge drinkers, BDCA = polyconsumers (BD + cannabis); PAS Family and PAS friends scores range between 10 and 50; PAS mother and PAS father score range between 12 and 60.
